# Differential trait responses of *Phragmites australis* to urbanisation

**DOI:** 10.1038/s41598-026-50177-y

**Published:** 2026-06-05

**Authors:** Viktor R. Tóth, Dorina Nagy

**Affiliations:** 1 HUN-REN, Balaton Limnological Research Institute, Klebelsberg Kuno utca 3, Tihany, H-8237 Hungary; 2 National Laboratory for Water Science and Water Security, HUN-REN, Balaton Limnological Research Institute, Klebelsberg Kuno utca 3, Tihany, H-8237 Hungary

**Keywords:** Hemeroby, Urbanisation, Littoral zone, Phenology, Reproductive, Vegetative, Ecology, Ecology, Environmental sciences

## Abstract

The littoral zones of shallow lakes are critical transitional habitats and emergent macrophytes, such as common reed (*Phragmites australis*), frequently dominate these margins. The potential effects of urbanisation on the common reed in the littoral zone of Lake Balaton were investigated. Elevated artificial night light levels were detected along the shoreline, reflecting extensive human activity, with littoral zones exhibiting significantly higher illumination than pelagic areas. No statistically significant correlations were observed between artificial night light and most vegetative parameters, while reproductive biomass and the proportion of flowering individuals were found to be significantly negatively correlated with it. Furthermore, a statistically significant positive correlation was identified between artificial night light and the timing of the start of the season, indicating a delayed initiation of seasonal growth in more urbanised sites. The findings reveal that urbanisation does not uniformly impair plant performance but acts as a complex environmental filter, shaping specific functional traits. Generative traits and the onset of seasonal growth appear more sensitive to urban pressures compared to vegetative growth, also emphasising the importance of integrating indicators for a more holistic assessment of urban impacts on aquatic vegetation.

## Introduction

 The littoral zones of shallow lakes are a critical transitional habitat between land and water^1^. These zones often support high biodiversity and underpin aquatic food webs^2–5^. In aquatic ecosystems, littoral habitats have been identified as key areas for food-web functioning. Emergent macrophytes - such as common reed (*Phragmites australis* (Cav.) Trin. ex Steud. – from now on *Phragmites*) - frequently dominate these shallow margins due to a wide range of competitive traits^6–8^. These plants perform multiple important ecosystem services^9–11^, providing also shelter, food and habitat for a wide range of species, further shaping the structure of the littoral zone.

Urbanisation induces profound changes in both terrestrial and aquatic plant communities through altered resource availability, novel environmental stressors, and disrupted ecological interactions^12–14^. These shifts represent strategic adaptations to urban pressures but often come at the cost of reduced biodiversity and ecosystem functionality^15,16^.

While urban plants do provide multiple ecosystem services for residents^17–19^, urbanisation is putting increasing pressure on natural ecosystems^20^. The history of human settlements, particularly in Europe, is marked by a complex network of of interconnected urban, peri-urban, and natural systems that still define the European landscape today. The process of urbanisation continues and is gradually reducing the amount of natural areas. Converting natural land for urban and suburban development increases the use of all kinds of impermeable surfaces and restricts the natural flow^21^. Meanwhile, runoff, wastewater and industrial discharges introduce high levels of nutrients and pollutants into adjacent bodies of water^22–24^. Another aspect of urbanisation is the creation of heat islands, i.e. areas where man-made surfaces absorb and retain heat, resulting in higher ambient temperatures than in the surrounding rural areas. This can elevate water surface temperatures at the shoreline^25^, thereby intensifying metabolic stress on littoral species^26^. Shallow littoral zones, with limited thermal inertia, experience extreme diurnal fluctuations - a key stressor for temperature-sensitive species and are favoured by cyanobacteria over macrophytes. Such anthropogenic inputs often drive eutrophication and can favour rapid vegetative growth and biomass accumulation. In urbanised wetlands, resilient, fast-growing macrophyte species (often including *Phragmites*) may proliferate, sometimes at the expense of other species.

*Phragmites* is a cosmopolitan emergent macrophyte of considerable ecological significance^27–29^. It is well adapted to a wide range of wetland conditions and often forms extensive stands in lake littoral zones. The common reed plays a crucial role in wetland structure and function: its dense roots and rhizomes stabilise sediment and influence hydrology, while its stiff stems provide habitat for fauna^27,30,31^. Though its role is gradually diminishing due to the lack of suitable sustainable harvesting technologies, *Phragmites* still holds economic (thatching and artisanal crafts) and cultural (heritage and community identity) significance in Central Europe.

Urbanisation can be considered from two distinct theoretical perspectives. Firstly, it can be considered a generalised ecological gradient, representing a spectrum ranging from natural to heavily modified environments^32^. Secondly, it can be regarded as a characteristic suite of disturbances from different human activities that alter the quality of the climate, soil, air and water in settlements and their surroundings^33^. Regardless of how urbanisation is viewed, satellite-derived artificial night lights (ANL) could be a powerful proxy for urbanisation intensity (hemeroby) in ecological studies^34,35^. Night-time radiance measured by sensors (e.g. the Visible Infrared Imaging Radiometer Suite Day/Night Band (VIIRS DNB) instrument) correlates strongly with human population density and economic activity. As such, ANL provides a spatially continuous index of human disturbance across landscapes. In the present study, ANL is used to quantify the degree of urban pressure on the lake’s littoral zone.

This study investigated the potential effects of urbanisation on the morphological and phenological parameters of common reed at the littoral zone of a shallow lake. The degree of urbanisation was approximated using satellite-derived ANL as a hemeroby indicator, i.e. as a proxy to the degree of human-induced deviation from natural ecosystems. The ANL was not used to estimate the effect of light pollution, but rather as a way to measure the combination of all human-induced changes. The hypothesis was that urbanisation would be associated with alterations in the morphological traits of common reed (*Phragmites australis*) in the littoral zone of a shallow lake. Specifically, it was expected that higher ANL levels would be associated with increased plant height, increased number of green leaves and increased stem biomass, reduced proportion of generative biomass and a lower proportion of flowering individuals. This expectation was based on the assumption that the combined effects of urbanisation, such as elevated temperatures associated with the urban heat island effect, increased artificial light availability and enhanced nutrient inputs, might extend diurnal and seasonal growth periods. These conditions could result in earlier leaf emergence and delayed leaf senescence compared to darker, less disturbed environments, favouring vegetative growth over reproductive investment.

**Fig. 1:**
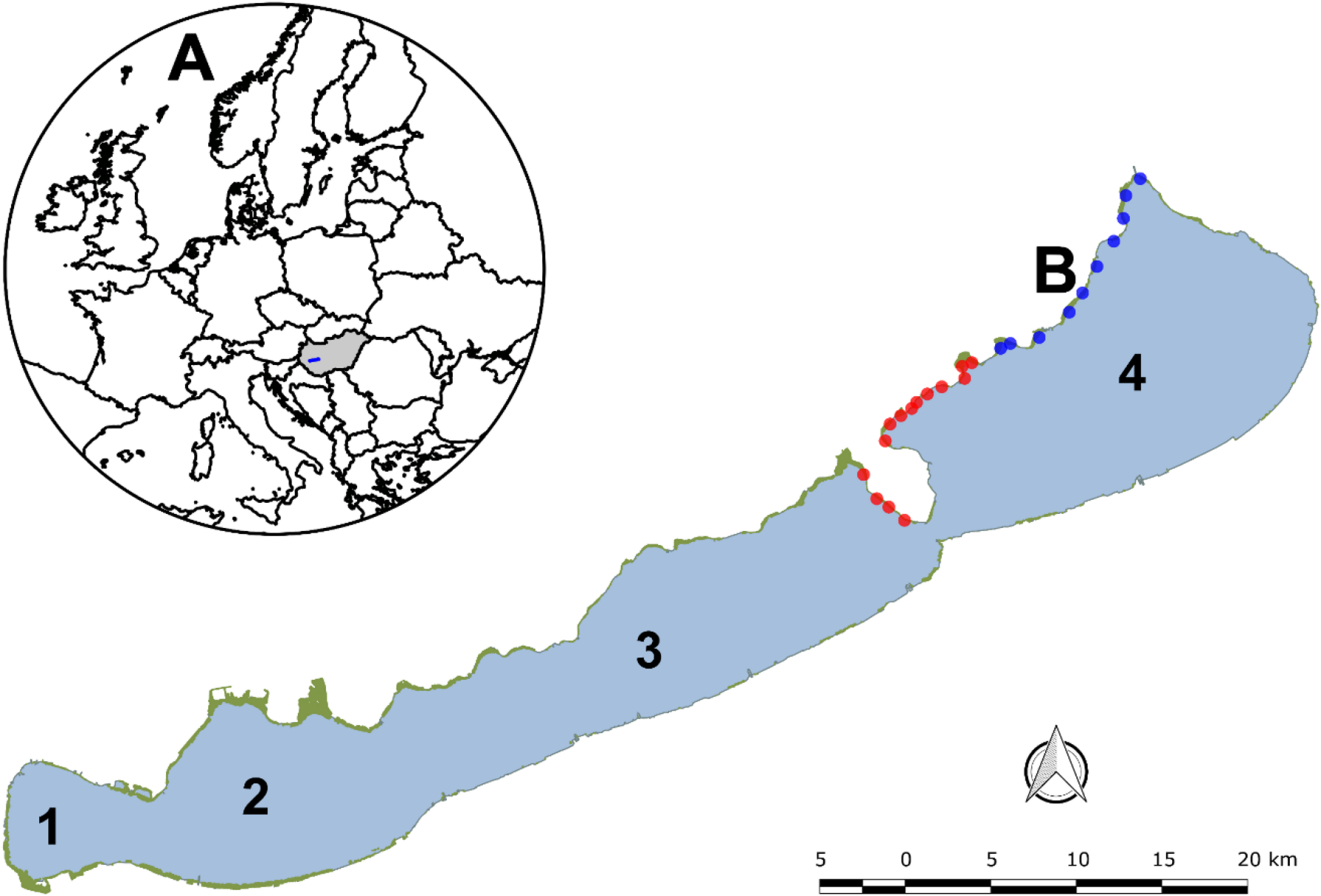
(**A**) - Map of Europe showing Hungary (grey) and Lake Balaton (blue polygon). (**B**) - Topographic map of Lake Balaton with sampling sites (blue dots – 2023, red dots − 2024) along the reed stands (green polygon) of the northern shore. Numbers show the basins of Lake Balaton. Images were created using QGIS 3.44 (https://qgis.org/download/).

## Methods

### Plant measurements

Samples of the common reed (*Phragmites australis* (Cav.) Trin. ex Steud.) were collected only from the northern shore of Lake Balaton (Fig. 1; Table 1). *Phragmites australis* plants were identified by the authors. Sampling was conducted during the initial flowering phase of the plants in early August of two consecutive years: 2023 and 2024. Ten sites were sampled in 2023, while fourteen sites were sampled in 2024 (Fig. 1B; Table 1). The decision to extend the sampling period into a second year was made because the 2023 dataset (from the first year) was believed to be insufficiently large due to the narrow time window required to capture the reproductive phase. As the hydrological and thermal conditions in 2023 and 2024 were highly similar, the second year provided an opportunity to take repeat measurements at additional sites in similar environmental conditions. This increased the robustness and representativeness of the dataset.

**Table 1:** Coordinates of the study sampling points in 2023 and 2024.

2023	2024
17.941990, 46.973981	46.92819, 17.86197
17.950430, 46.976558	46.93297, 17.86138
17.970486, 46.981985	46.93713, 17.86645
17.989883, 46.992148	46.94096, 17.87402
18.001270, 47.000856	46.94878, 17.88509
18.009195, 47.017009	46.95489, 17.89864
18.028393, 47.042634	46.95463, 17.90815
18.027792, 47.042556	46.95944, 17.91640
18.029680, 47.048872	46.96477, 17.91715
18.040695, 47.057722	46.96638, 17.92315
	46.89262, 17.87239
	46.89839, 17.86128
	46.90300, 17.85085*
	46.90961, 17.84868

* - point at which the voucher specimen was sampled. A specimen was deposited in the herbarium of the Bakony Natural History Museum at the Hungarian Natural History Museum (reference number is HNHM-BMVAS 1601).

Sampling was performed from a boat. Sampling sites were selected using a quasi-random approach to ensure that certain ecological and spatial criteria were met. Each site represented a stable, monospecific reed stand with no visible signs of degradation. The effects of shading caused by riparian vegetation or artificial structures were avoided. Sampling points were positioned within the central part of reed stands, extending at least 50 m along the shoreline, to minimise edge effects. All sites were located in the eastern part of the lake, where environmental conditions are generally oligo-mesotrophic and relatively uniform across the northern shore of the basin, though no sediment nutrient data were available. Overall, the sites were not equidistant, with the shortest distance between two sites being approximately 490 m and the greatest separation being around 2.7 km. At each sampling site, fifteen individual reed plants within 2–3 m were randomly collected cutting them at water surface level with a pair of pruning shears. These plants were consistently collected 2 m into the reed stands along the waterfront side. Thus, at each site, plants were collected at or near their maximum depth of distribution. Water depth was recorded at each sampling point and added to the measured plant height to obtain total shoot length, but depth itself was not treated as an independent parameter in the analyses.

Following collection, a series of morphological trait measurements were performed on each plant. The total length of each plant was measured from its base to the tip of the longest leaf or inflorescence using a tape measure with centimetre precision. Afterwards, the measured water depth at each site was added to the height of the plants. The diameter of the basal internode was recorded using a vernier calliper with a precision of 0.1 mm. The number of green leaves and the total number of nodes along the stem were also counted. Finally, the presence or absence of an inflorescence was noted for each plant.

Once the morphological trait measurements had been completed, each plant was carefully divided into its constituent parts: stems, leaves and inflorescences. These parts were then placed in a drying oven set at 65 °C. Once the plant parts had dried to a constant weight, they were weighed individually using a calibrated analytical balance.

### Artificial night light estimation

For the estimation of ANL, the monthly average VIIRS DNB images that were closest to the data sampling period were acquired (October 2023 and 2024). These data were obtained from The Earth Observation Group (EOG), Payne Institute for Public Policy, Colorado School of Mines: https://eogdata.mines.edu/products/vnl/.

**Fig. 2:**
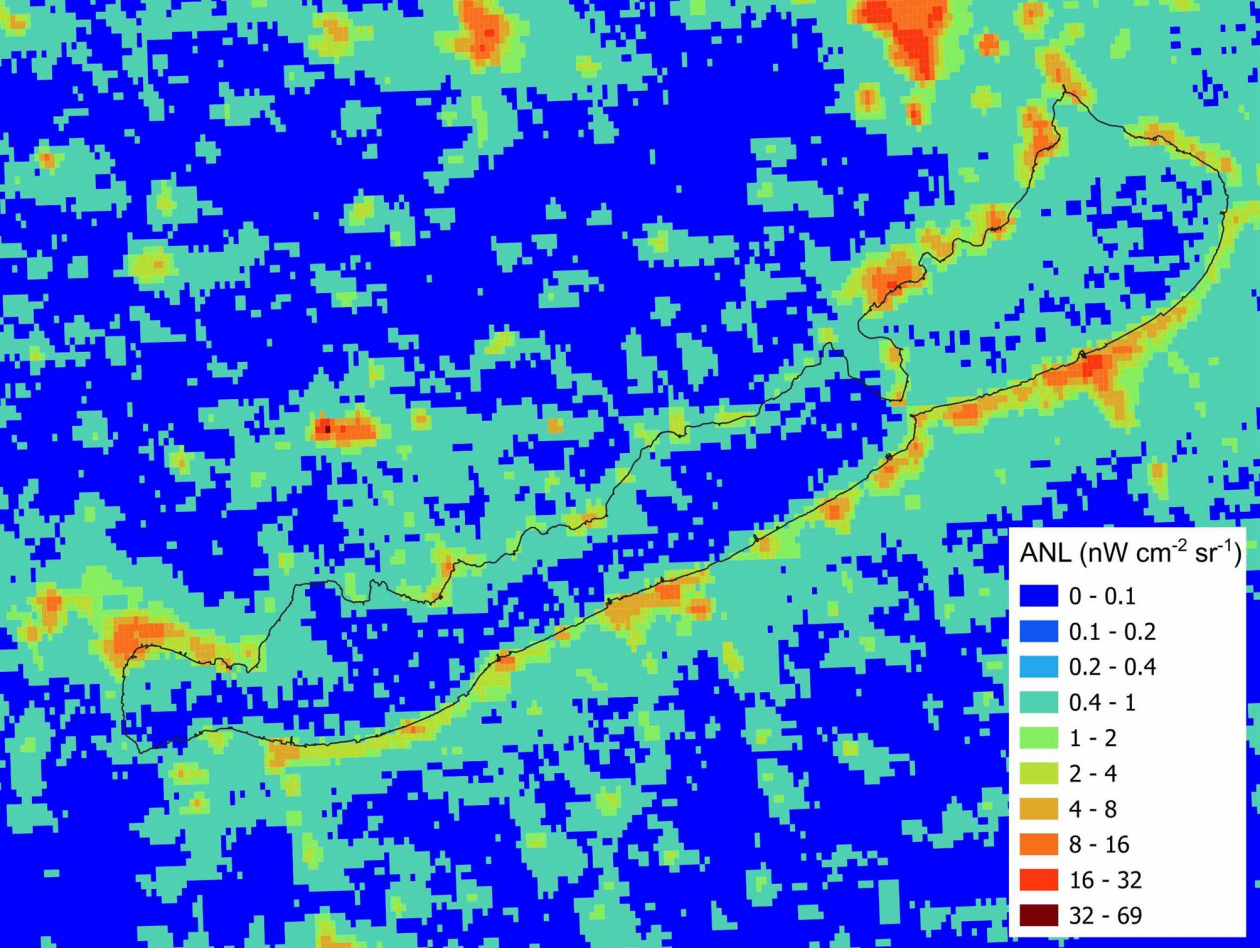
Artificial night light (ANL) derived from the Visible Infrared Imaging Radiometer Suite (VIIRS) Day/Night Band (DNB) satellite image of Lake Balaton and its surroundings from October 2023. Image was created using SNAP v12 and QGIS 3.44 (https://qgis.org/download/ and https://step.esa.int/main/download/snap-download/2/).

The retrieval of ANL data in form of nW cm^− 2^ sr^− 1^ from VIIRS DNB satellite images was performed using the GPS coordinates of the sampling sites. Data from 2023 October are shown on Fig. 2.

### Phenological assessment of the study sites

Normalised Difference Vegetation Index (NDVI) data for the studied sites were acquired via Google Earth Engine using surface reflectance products from Landsat 8 and 9 satellites. Only cloud-free scenes from 2023 to 2024 were selected for analysis. The extracted NDVI time series were then imported into TIMESAT software (version 3.3; https://web.nateko.lu.se/timesat/timesat.asp) for phenological analysis.

Within TIMESAT, an asymmetric Gaussian fitting method was applied to smooth the NDVI time series and extract key phenological metrics.

The major phenological parameters were derived as follows:

- The length of the season (LoS).
- The peak of the season (PoS).
- The peak NDVI value (Peak).
- The NDVI amplitude (Amp).
- The start of the season (SoS).
- The end of the season (EoS).

The SoS and EoS were determined as 20% higher than the 2-year NDVI baseline. This threshold was chosen because it best matched our in situ observations of reed phenology. The seasonality data were then exported for further statistical analysis.

### Statistical analysis

All morphological trait measurements are presented as the average ± standard deviation (*n* = 15). To assess the relationship between ANL and morphological, biomass-related and phenological parameters of *Phragmites* correlation analysis was performed using the R statistical software environment version 4.3.2^36^. Spearman’s rank correlation was used as this non-parametric method is better suited to seemingly non-linear relationships and does not require normalisation of the data. Furthermore, Spearman’s rank order correlation is less sensitive to outliers than the Pearson correlation, making false positive correlations less likely.

The parameters were compared using independent samples t-tests and Mann-Whitney U test. The assumptions of normality and homoscedasticity were assessed when it was necessary. Where deviations from normal distribution or unequal variances were detected, appropriate data transformations (e.g. log or square root) were applied to meet the test assumptions.

## Results

The study used daily average water level data for Lake Balaton, which was collected by the Central Transdanubian Water Directorate and organised by the General Directorate of Water Management of Hungary (https://www.hydroinfo.hu/vituki/archivum/ba.htm). In August 2023, the mean water level of Lake Balaton was recorded as 3.52 ± 0.03 m. In August 2024, this value was slightly lower at 3.48 ± 0.03 m. Air and water temperature data for 2023 and 2024 were obtained from the Hungarian Meteorological Service (Hungaromet) for the easternmost, Siófok basin of Lake Balaton and compared between 2023 and 2024. The mean annual air temperatures were found to be 13.3 ± 8.1 °C and 14.1 ± 8.8 °C, respectively (Mann-Whitney U = 63 601.5, *P* = 0.263). Similarly, the mean annual water temperatures were determined to be 13.5 ± 8.4 °C and 13.8 ± 8.5 °C (Mann-Whitney U = 64 814.0, *P* = 0.488). These values reflect the relatively stable hydrological and meteorological conditions during 2023 and 2024, and demonstrate the intercomparability of the morphological data between the two years.

Considerable spatial heterogeneity was observed across the lake surface, indicating high heterogeneity and specific light patterns (Fig. 2). Elevated ANL levels were detected along the shoreline, where pixel radiance values ranged between 1 and 16 nW cm^− 2^ sr^− 1^, reflecting extensive human activity and shoreline development (Fig. 2). Moreover, major urban centres around the lake had even higher radiance levels, reaching 16–24 nW cm^− 2^ sr^− 1^. In contrast, the pelagic regions of the lake showed much lower ANL intensities, typically between 0.1 and 2 nW cm^− 2^ sr^− 1^.

The morphology of *Phragmites* plants sampled in 2023 and 2024 along the northern shore of Lake Balaton was characterised by a mean plant height of 391.0 ± 51.7 cm. The mean diameter of the basal internode was 9.2 ± 1.4 mm. There was an average of 16.3 ± 2.5 green leaves per plant, associated with a total of 20.1 ± 3.5 nodes. The mean above-water dry biomass per plant was 37.0 ± 13.3 g, of which 30.5 ± 5.2% was photosynthetically active tissue (leaves) and 0.8 ± 0.9% was reproductive structures (inflorescences).

**Fig. 3:**
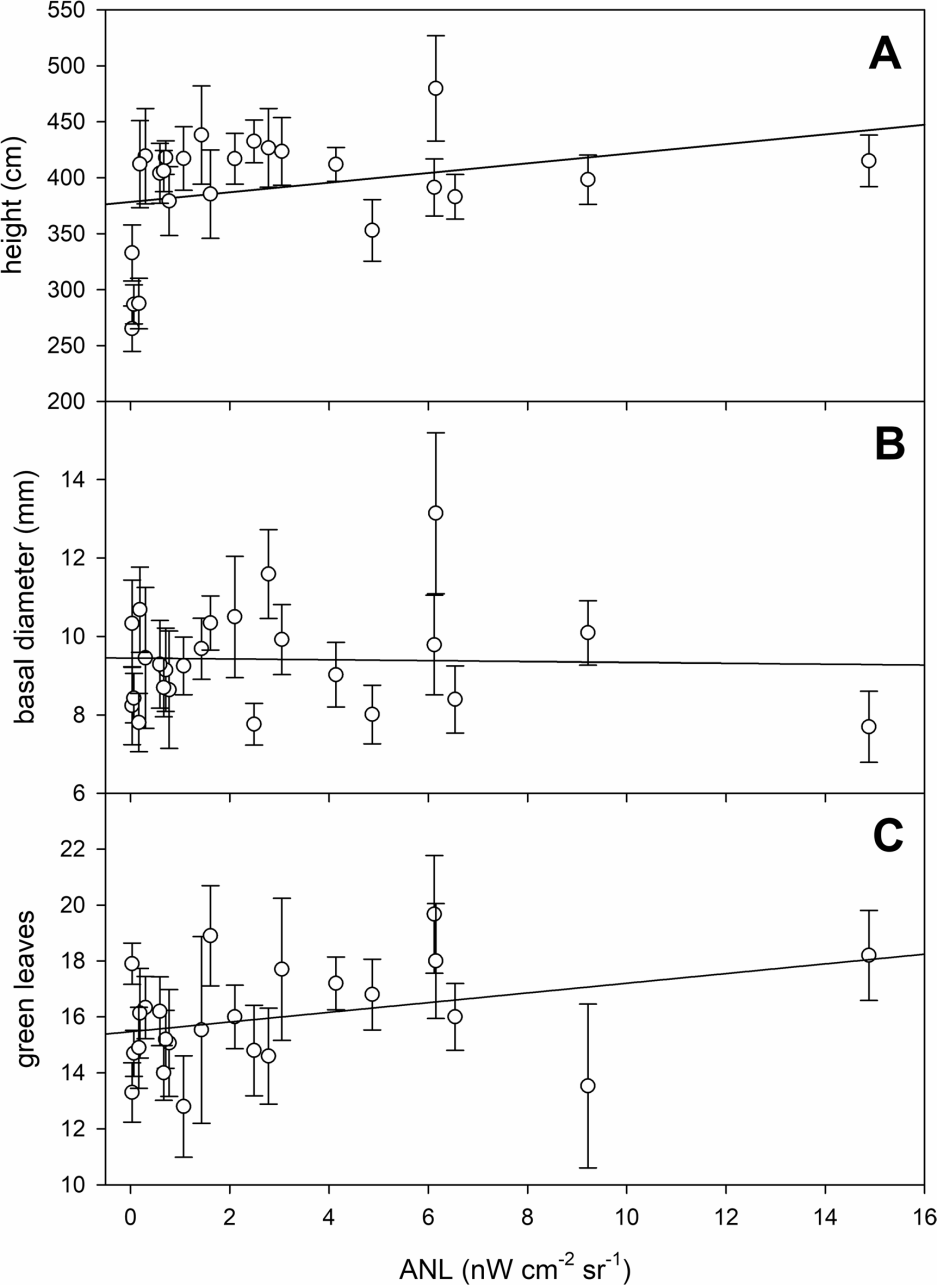
Change of the major morphological parameters (plant height – (**A**), basal diameter - (**B**), and number of green leaves - (**C**) of *Phragmites australis* plants at the study sites along the reed stands of the northern shore of Lake Balaton. Each symbol shows average ± SD of 15 samples. The solid line shows the linear regression fitted to the data and is intended solely as a visual guide.

Plant height showed considerable (84%) variation in all the studied plants, ranging from 265.1 ± 20.3 cm to 479.9 ± 47.0 cm (Fig. 3A). The lowest mean height was recorded at a site with an ANL of 0.03 nW cm^− 2^ sr^− 1^, while the maximum height was observed at a site with an ANL of 6.15 nW cm^− 2^ sr^− 1^. Basal internode diameter of *Phragmites* plants also showed significant variation as it ranged from 7.7 ± 0.9 mm to 13.1 ± 2.1 mm, with the smallest diameter recorded at a site with an ANL of 14.88 nW cm^− 2^ sr^− 1^ and the largest at a site with an ANL of 6.15 nW cm^− 2^ sr^− 1^ (Fig. 3B). The number of green leaves per plant varied between 12.8 ± 1.8 and 19.7 ± 2.1, with the lowest mean recorded at a site with an ANL of 1.07 nW cm^− 2^ sr^− 1^ and the highest at a site with an ANL of 6.12 nW cm^− 2^ sr^− 1^ (Fig. 3C).

**Fig. 4:**
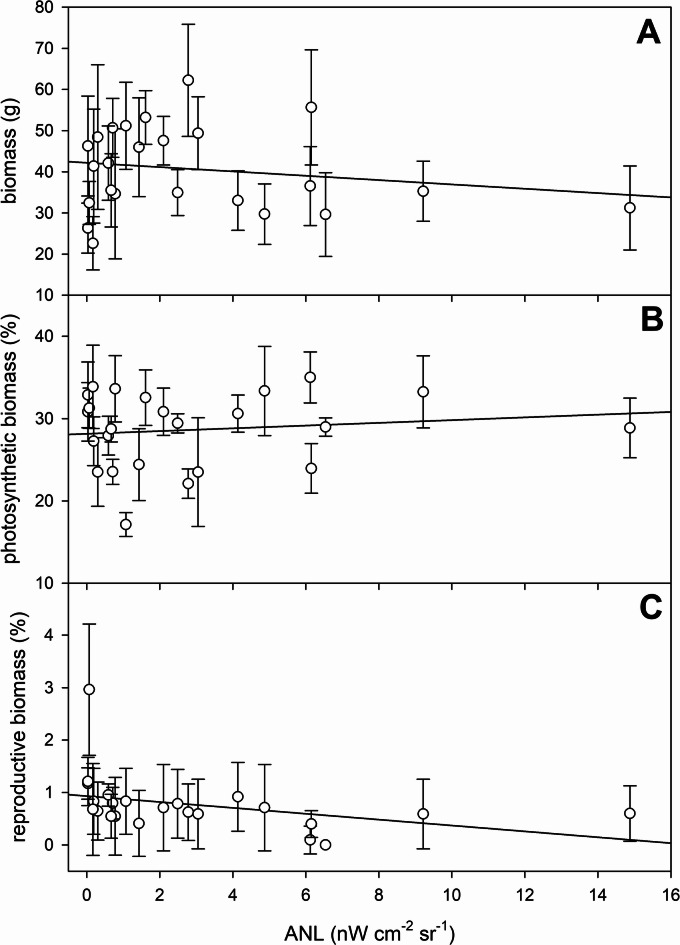
Change of the biomass parameters (total biomass – (**A**), photosynthetic biomass (as % of total biomass) - (**B**), and reproductive biomass (as % of total biomass) - (**C**) of *Phragmites australis* plants at the study sites along the northern shore of Lake Balaton. Each symbol shows average ± SD of 15 samples. The solid line shows the linear regression fitted to the data and is intended solely as a visual guide.

**Fig. 5:**
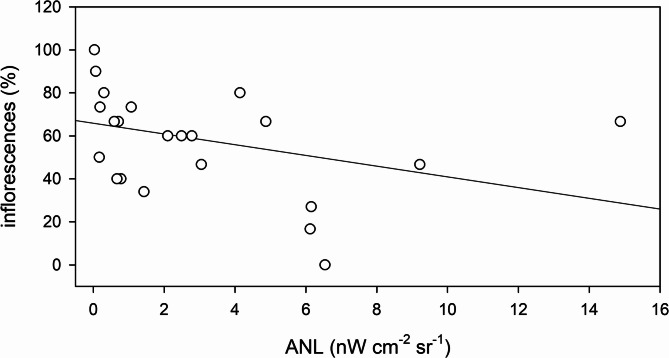
Ratio of *Phragmites australis* plants with inflorescences (as a % of total plants) at the study sites along the northern shore of Lake Balaton. The solid line shows the linear regression fitted to the data and is intended solely as a visual guide.

Total dry biomass per plant varied between 22.62 ± 6.47 g (ANL = 0.17 nW cm^− 2^ sr^− 1^) and 62.23 ± 13.62 g (ANL = 2.78 nW cm^− 2^ sr^− 1^), i.e. the largest plants were 175% bigger than the plants from the site with smallest plants (Fig. 4A). The variation of the photosynthetically active tissue (leaves) was 104%, as the proportion of photosynthetic biomass ranged from 17.14 ± 1.45% (ANL = 1.07 nW cm^− 2^ sr^− 1^) to 35.01 ± 3.10% (ANL = 6.12 nW cm^− 2^ sr^− 1^) of the total biomass (Fig. 4B). Reproductive biomass, expressed as a percentage of total aboveground biomass, ranged from 0 ± 0% at the site with ANL of 6.54 nW cm^− 2^ sr^− 1^ to 2.96 ± 1.25% at an ANL of 0.07 nW cm^− 2^ sr^− 1^ (Fig. 4C). The percentage of flowering individuals at each site was observed to decrease from full flowering (100%) at low light sites (ANL = 0.03 nW cm^− 2^ sr^− 1^) to no flowering (0%) at the ANL site of 6.54 nW cm^− 2^ sr^− 1^ (Fig. 5).

**Table 2:** Spearman’s rank correlation coefficients (ρ) and significance values (p) between artificial night light and morphological and biomass parameters of *Phragmites australis* plants sampled along the northern shore of Lake Balaton.

	ρ	*P*
Height	0.355	0.087
Basal diameter	0.058	0.787
Green leaves	0.331	0.114
Nodes	0.344	0.099
Total biomass	0.031	0.885
Photosynthetic biomass	-0.018	0.934
Reproductive biomass	-0.634	0.001
Inflorescence	-0.573	0.004

No significant correlations were found between ANL and plant height, basal diameter, number of green leaves, number of nodes, total biomass or photosynthetic biomass (Figs. 3 and 4; Table 2). In contrast, reproductive biomass (Fig. 4C; Table 2) and the presence of inflorescences (Fig. 5; Table 2) were significantly negatively correlated with ANL.

**Fig. 6:**
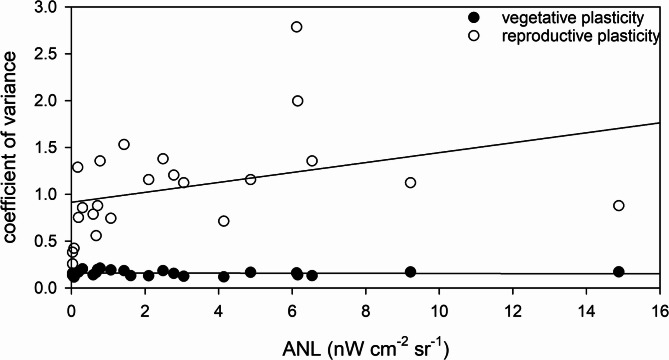
Vegetative and generative plasticity calculated as coefficient of variance of *Phragmites australis* plants at the study sites along the northern shore of Lake Balaton. The solid lines show the linear regression fitted to the data and are intended solely as a visual guide.

Figure 6 shows that *Phragmites* plants respond differently to urbanisation in terms of vegetative and generative plasticity. Vegetative plasticity exhibited relatively low variability (0.161 ± 0.089) and showed no significant correlation with ANL (Spearman rank order correlation: ρ = -0.081, *p* = 0.707). Generative plasticity, however, was significantly higher (1.039 ± 0.655; Kruskal-Wallis test: H = 34.5, *p* = 4.2 × 10^− 9^) and demonstrated a statistically significant positive correlation with ANL (Spearman rank order correlation: ρ = 0.574, *p* = 0.004) (Fig. 6).

Based on the NDVI time series, the average start of the season (SoS) for *Phragmites australis* at the studied sites was identified as 7 April (± 5 days), while the end of the season (EoS) occurred on 9 November (± 4 days). Accordingly, the average length of the growing season (LoS) was estimated to be 216 days (± 7 days). The peak of the season (PoS), as indicated by maximum NDVI values, was observed on 31 July (± 3 days), with a mean NDVI of 0.45 (± 0.09).

The relationship between artificial night light (ANL) intensity and the derived phenological parameters was assessed using Spearman’s rank correlation (Table 3). A significant positive correlation was found between ANL and the timing of the start of the season (ρ = 0.454, *p* = 0.026, indicating a gradual delay in its onset. The difference between the earliest and latest recorded start dates was eight days. No statistically significant correlations were observed for other phenological parameters (Table 3).

**Table 3:** Spearman’s rank correlation coefficients (ρ) and significance values (p) between artificial night light and phenological parameters of *Phragmites australis* plants sampled along the northern shore of Lake Balaton.

	ρ	*p*
Start of the season	0.454	0.026
End of the season	0.233	0.269
Length of the season	-0.186	0.385
Peak of the season	0.279	0.187
NDVI peak value	0.306	0.146
NDVI amplitude	0.079	0.713

## Discussion

Freshwater lakes used intensively for recreation face unprecedented pressure from surrounding development. This fundamentally alters their littoral zones and the aquatic plant communities that depend on these critical habitats^37^. Research has revealed a consistent pattern of ecological degradation driven by urbanisation, resulting in significant changes to water quality, habitat structure, and plant biodiversity in shallow nearshore areas, which traditionally support the most diverse aquatic ecosystems^38,39^. These changes have implications not only for the local environment, but also for broader shifts in ecosystem functioning. This affects processes such as carbon cycling and water quality regulation, as well as biodiversity conservation in urban landscapes^40^.

Urbanisation transforms the littoral zones of lakes through various processes of physical and chemical restructuring and biological invasion, thus disrupting the aquatic environments and communities that are critical to lake ecosystems. This study investigated the potential effects of urbanisation on the morphological and phenological parameters of common reed in the littoral zone of a shallow lake. Satellite-derived artificial night light (ANL) was used as an indicator of hemeroby, i.e. as a proxy for the degree of human-induced deviation from natural ecosystems.

The results of the present study provide a nuanced picture, offering strong support for some parts of the hypothesis but not others. Strong evidence was found in support of the hypothesised effects on reproduction, as a negative correlation was identified between ANL levels and both reproductive biomass and the occurrence of inflorescences, while a positive correlation was detected between ANL levels and the start of the season in *Phragmites* plants. This negative relationship suggests that delayed or suppressed flowering may be a more widespread response to urbanisation, particularly in warmer temperate or Mediterranean regions^41–44^. Although earlier and more prolific flowering has often been reported in urban contexts^41–44^, these trends appear to be less consistent among late summer-flowering species, such as *Phragmites*. Previous studies have demonstrated that urbanisation can exert variable effects on different flowering stages, advancing some while delaying others^43^, and that the magnitude and direction of these shifts depend on the climate^45^. In warmer regions such as the Lake Balaton area, urban-induced warming and prolonged photoperiods may disrupt the internal regulatory mechanisms of photoperiod-sensitive species rather than advance flowering, potentially leading to delayed or incomplete reproductive development. Thus, findings of this study suggest that urbanisation’s influence on phenology is not universal, but is instead modulated by plant functional traits, species-specific sensitivity and regional climatic conditions. Consequently, the later or reduced flowering observed in *Phragmites* may be a manifestation of the broader ecological response of wetland plants to urban-driven environmental change.

The absence of statistically significant relationships between ANL and the majority of the vegetative parameters of *Phragmites* plants, including plant height, basal diameter, photosynthetic biomass and total biomass, suggests that urbanisation, as represented by artificial illumination, does not have a significant or consistent impact on the structural growth of this species within the littoral wetland environment under study. Although weak positive trends were observed in the number of green leaves and internodes, these did not reach statistical significance. This suggests that any vegetative response to urban pressure may be minor, context-dependent or obscured by other environmental variables. This outcome reflects the broader complexity of urbanisation’s impact on plant growth. While terrestrial systems often exhibit biomass reductions due to habitat loss and fragmentation^46,47^, aquatic and semi-aquatic systems respond in more variable ways. In such habitats, urban pressures can lead to reductions in biomass via physical disturbance and pollution, and to increases in biomass, particularly when nutrient enrichment favours the proliferation of fast-growing, opportunistic species, although excessive enrichment can also result in shifts in the ecosystem that disadvantage macrophytes in favour of phytoplankton^47–49^.

Interpreting trait responses based exclusively on a single urbanisation proxy presents inherent limitations. While ANL functions effectively as a continuous index of hemeroby, reflecting a combination of human-induced disturbances, isolating specific causal factors is challenging. Key environmental co-variables known to modulate *Phragmites* growth and morphology, including sediment nutrient availability, general and local hydrology, were not included in the analysis. Although water depth was recorded at each sampling point, it was not used as an independent analytical parameter. The absence of statistically significant correlations between ANL and many vegetative parameters (e.g. plant height and total biomass) suggests that these parameters are regulated by other local stressors that were not measured in the present study rather than by the overall urban gradient captured by ANL. Taken together, these data highlights the importance of evaluating multiple environmental stressors together, rather than attributing biomass or morphological changes to a single urbanisation proxy.

This study showed that the generative traits of *Phragmites*, such as the development and allocation of resources to reproductive structures, are more responsive to urban pressures than the vegetative traits of height, biomass or leaf number. The lack of significant variation in vegetative plasticity across the urbanisation gradient may suggest greater functional stability in vegetative development. In a similar manner, uniform plasticity was exhibited in the photophysiological traits of *Phragmites* plants along a degradation gradient, albeit to a greater extent^50^. Conversely, the increased generative plasticity and its positive correlation with ANL suggest that reproductive strategies are more adaptable and sensitive to environmental heterogeneity caused by urbanisation. This differential response may reflect the ecological costs and trade-offs associated with reproduction under anthropogenic stress. At more urbanised or disturbed sites, increased plasticity in reproductive traits could suggest the destabilisation or suppression of reproductive development in response to the suboptimal environmental conditions typical of urban areas.

The growth and senescence cycles of emergent plants at lake shores can also be quantified using time series of NDVI data^8,51,52^. The average onset of the growing season was detected in early April, with termination occurring in early November. This defines a typical growing season length of 216 days. The maximum NDVI value was recorded at the end of July, suggesting a mid-summer productivity peak characteristic of temperate reed stands. The only statistically significant positive correlation was between ANL levels and the timing of the start date of the growing season (SoS), indicating that urbanisation is associated with delayed initiation of seasonal growth. Higher levels of urbanisation are associated with a greater delay, with a maximum delay of eight days. Although no comparable studies of emergent macrophytes were found, ANL tended to advance SoS in terrestrial vegetation^53,54^. However, the overall response of SoS to ANL was relatively weak compared to that of other potential factors. This supports the idea that phenological responses are integrative indicators of environmental conditions^5,55^. *Phragmites* plants in more disturbed sites begin their growing season later, possibly due to cumulative suboptimal conditions. Furthermore, these data imply that the impact of urbanisation is concentrated in the early stages of growth, while later stages may be more resilient, potentially due to compensatory growth or adaptive mechanisms.

This study sheds new light on the ecological responses of *Phragmites* to urbanisation in littoral zones of a large, shallow lake by using artificial night lighting as a proxy for hemeroby. The findings reveal the complexity of urban impacts on aquatic macrophytes and show that sensitivity varies across different trait categories. Furthermore, the study revealed that urbanisation was associated with a delayed onset of the growing season, suggesting that the early stages of development are more susceptible to disturbance, potentially due to cumulative anthropogenic stressors. Together, these findings emphasise that urbanisation does not uniformly impair plant performance, but instead acts as a complex environmental filter that shapes specific functional traits. The combination of trait- and phenological-level responses reflects the nuanced ways in which *Phragmites* adapts to human-modified habitats, with implications for its persistence and ecological function in changing littoral systems. These results emphasise the importance of integrating morphological, reproductive and temporal indicators when assessing urban impacts on aquatic vegetation, and highlight the need for more holistic approaches to monitoring and managing urban shorelines in freshwater ecosystems. Future in situ research should aim to disentangle the individual and interactive effects of urbanisation-related factors, such as artificial light, nutrient enrichment, and hydrological alteration, on aquatic plants of the littoral zone, ideally combining physiological, morphological, and community-level approaches across multiple spatial and temporal scales.

## Data Availability

Morphological data will be available for non-commercial purposes after acceptance of the manuscript in the repository of the Hungarian Research Network (https://researchdata.hu/en). For data please contact Viktor Tóth.
